# Post-traumatic stress disorder among tunisian healthcare professionals facing the pandemic coronavirus (COVID-19)

**DOI:** 10.1192/j.eurpsy.2021.835

**Published:** 2021-08-13

**Authors:** L. Aribi, R. Jbir, N. Messedi, D. Gdoura, W. Bouattour, N. Kolsi, F. Charfeddine, J. Aloulou

**Affiliations:** 1 Psychiatry, Hedi Chaker Sfax, Sfax, Tunisia; 2 Psychiatry, Hedi chaker Sfax, sfax, Tunisia; 3 Psychiatry (b), Hedi Chaker University hospital, sfax, Tunisia

**Keywords:** Post traumatic Stress Disorder, COVID-19, health professionals

## Abstract

**Introduction:**

The new coronavirus has spread rapidly across the planet confining entire populations, filling hospitals overwhelmed by massive arrivals of patients This new health situation was traumatic especially for health-professionals

**Objectives:**

To study the prevalence and predictors of post-traumatic stress disorder, among health-workers.

**Methods:**

Our study was descriptive and analytical cross-sectional, between May until June 2020. An anonymous online-survey was sent to collect those parameters Sociodemographic-information Physical symptoms The existence of contact with a suspected case The need for quarantine The stressful event The state of mental health, using: PCL-5: 20 items which measures the 20 symptoms of post-traumatic stress-disorder according to DSM-5. PSQI: 9 questions to see the existence or not of a disturbance in sleep

**Results:**

125 participants: 28 university-hospital doctors, 55 residents, 5 interns, 4 specialist-doctors, 2 general-practitioners, 14 nurses, 14 senior-technicians, 2 midwives and a pharmacist. The average seniority at the job was 6 years. Two factors were the most stressful: The characteristics of this pandemic 37.6% The fear of caching the virus and transmit it to their families: 37.6%. 42.4% of participants presented a post-traumatic stress disorder. 3 parameters were correlated with post-traumatic stress disorder: young age, having children (p = 0.007) and fewer years of professional-experience. This pandemic altered the quality of sleep of caregivers, 62.4% of them had a bad quality of sleep. The bad sleepers developed more post-traumatic stress disorder

**Conclusions:**

This health crisis had a major impact on the mental health of our heroes that is why we should provide them with the necessary psychological support.

